# Meningitis: A Suggestion of a New Treatment with Old Remedies

**Published:** 1907-06

**Authors:** R. R. Kime

**Affiliations:** Atlanta, Ga.


### *MENINGITIS: A SUGGESTION OF A NEW TREATMENT WITH OLD REMEDIES.**

By R. R. Kime, M.D.

In presenting the report of these cases to the Fulton County Medical Society, I desire to simply suggest a line of treatment for trial by others with a hope that it may result in good.

Last year at a discussion on meningitis by this society it was demonstrated the hopelessness of treatment in these cases set me to thinking. Reasoning from the effect of Norwood's Tr. Ver. Ver. in high temperature, high arterial tension and convulsions in children, its lessening of arterial tension, preventing consulsions, lowering temperature and preventing brain complications, also its effect in puerperal convulsions as well as venous section in these to overcome the invasion by the disease or germs.

It is also probable that the removal of 16 to 24 ounces of blood more or less according to condition of patient, will remove as many germs and toxines as the withdrawal of any amount of spinal fluid that can be done with safety.

Drainage from the brain can not be used successfully, as the point of effusion or disease can not be localized at first, later structural changes have taken place and the fluid will be too thick to drain off and there may be many foci of infection, then it would be impossible to reach all even if they could be drained.

Surgery was suggested and discussed at that meeting, but it occurred to me that veratrum and bleeding would accomplish all or more than surgery could do. By the judicious use of these remedies you can reduce the arterial tension, check inflammation, prevent effusion and bring pulse below normal and hold it there without detriment or harm to the patient. They will relieve arterial tension and slow the pulse as certain as 2 and 2 makes 4—nothing more certain in medicine.

Reasoning that these effects would certainly check effusion, relieve tension and give nature a chance.

---

*Read before Fulton County Medical Society, May 16th, 1907.

Case I. Male, age 60. Taken late in the evening. Dr. Kelley was called, saw him about 9:30 p. m., and diagnosed case as meningitis, but did not have medicines with him.

I saw case at 10:30 p. m. and concurred in diagnosis.

Patient suffering intensely with pain in back of head, etc.,—muscles rigid, in extremities and back of neck intense pain on movement, great difficulty in breathing owing to involvement of muscles of throat, had to be propped up in bed.

Pulse above 100, arterial tension high, patient previously had weak heart, possibly some sclerosis and kidney disturbance.

Abstracted 20 ounces blood—pulse and gave Tr. Ver. Ver. Nor. 6 gtts. Hyp., pulse came down rather quickly to 60 to 70 to min., some nausea, trying to vomit, some dimness of vision, gave Hyp. Morph., 1-3 gr. Dig. 1-100.

Case II. Age 35, married, weight 165 lbs.

Called March 31st, 11:30 a. m., patient not feeling well day previous, also this a. m., commenced with headache about 8 a. m., by 9:30 a. m. very severe, took a migraine tablet at 10:30, was delirious, uncontrolable, by 11 a. m. screaming, fighting, kicking, difficult to keep on bed.

At 11:30 a. m. gave Hyp. Morph 1-4 gr., Atroph. 1-150, Hyoscine 1-100, in about 30 minutes became more quiet, yet restless, unconscious, muscular twitchings, head thrown back, slight rigidity muscles, unconscious to prick of pin, eyes red, congested, pulse about 115 to 120, temperature could not get.

12:55 she was given veratrium gtts. iv., Ergotoee gtts. viii, Bromides z5s.

1:45 p. m. gave Tr. Ver Ver. Nor. gtts X Hyp.

2:15 p. m. symptoms the same except pulse fuller and 125 to 130 per min. Bled to amount of 24 oz., pulse immediately fell to 88, then in 30 mints. to 80, semi-consciousness. Ordered the Ver. Ver., Ergotole and Bromide srepeated every 1 to 2 hrs. until pulse below 70.

At 5:30 pulse 60, after that held from 50 to 60 for four days, by repeating Ver. Ver. Mixture from 2 to 6 hrs. apart.

Patient does not remember anything that transpired on Sunday,

Monday and Tuesday, complained some of pain in head on Tuesday, swimming, dizzy condition Wednesday and vision not clear until Thursday.

Congested condition of eyes cleared up shortly after bleeding, when pulse came to normal and below.

Before morphia was given, pain and rigidity of muscles almost disappeared, breathing better. After this the patient was given Ver. Ergot and Bromides combined with a diuretic.

Calomel and salines given—no food first 24 hours.

Pulse held at or below normal for several days, patient practically well in one week and I made but the one visit.

Remarks.—This treatment to be successful must be given heroically within first few hours of attack, before effusion and structural changes in brain or meninges have taken place.

The amount of blood removed depends on age, condition and vitality of patient, and must be governed by the effect on pulse and arterial tension. If too much blood is removed, then saline infusion can be used as needed.

It is essential to rouse all the secretions and hold the pulse below normal until patient is out of danger. I hope physicians will give this method of treatment a trial and report results to see if it will accomplish what is desired in the control of this dread disease.

DISCUSSION OF DR. KIME'S PAPER.

Dr. W. S. Goldsmith thought Dr. Kime's report timely and valuable. He, too, had recently heard of a patient who had been treated by bleeding and blistering and recovered satisfactorily.

Dr. Olmstead was reminded by the revival of venesection of the success of Dr. John G. Westmoreland, who had remarkable success in the treatment of miningitis by bleeding, and he thought this plan, combined with veratrum might prove valuable.

Dr. Wesley Taylor treated his patients somewhat along this line, but believed Dr. Kime rather courageous to withdraw as much as 20 ounces of blood from an aged patient. He considered his results remarkable if there was no doubt as to the diagnosis and this did not seem likely, the symptoms of the last case being almost typical.

Dr. Cyrus Strickler reported a case treated in the Grady Hospital while an interne who had been comatose for 24 hours with a pulse of 180 who was promptly benefited by veratrum. The pulse dropped to 120 and the patient was able to speak and say that he was better, but he finally died. Dr. Strickler said that he had had no experience with bleeding, but hoped Dr. Kime would continue this line of treatment. He believes the withdrawal of the spinal fluid is a rational procedure.

Dr. Kime urged that this treatment be carried out within the first 24 to 36 hours. As to the extraction of blood he thought that if a little too much were drawn an infusion of normal salt solution could easily be given to counteract any deleterious effects. The pulse, rate of bleeding should be carefully watched.

Dr. C. P. Ward sent a patient to the Grady Hospital recently and 3 ounces of fluid was withdrawn from his spine with prompt, but not permanent results.

